# Multiscale Feature Enhancement and Bidirectional Temporal Dependency Networks for Arrhythmia Classification

**DOI:** 10.3390/biology15020149

**Published:** 2026-01-14

**Authors:** Liuwang Yang, Chen Wang, Wenjing Chu, Hongliang Chen, Chuquan Wu, Yunfan Chen, Xiangkui Wan

**Affiliations:** 1 Hubei Key Laboratory for High-Efficiency Utilization of Solar Energy and Operation Control of Energy Storage System, Hubei University of Technology, Wuhan 430068, China; 102310261@hbut.edu.cn (L.Y.); 2310211129@hbut.edu.cn (C.W.); 15826821662@163.com (W.C.); 102410252@hbut.edu.cn (H.C.); yfchen@hbut.edu.cn (Y.C.); 2 Puleap (Wuhan) Medical Technology Co., Ltd., Wuhan 430000, China; wuchuquan@puleap.com

**Keywords:** arrhythmia classification, electrocardiogram, multiscale feature enhancement, multi-head self-attention mechanism, bidirectional temporal dependency

## Abstract

Cardiac arrhythmias, especially premature beats and atrial fibrillation, pose substantial clinical risks and detection hurdles. While deep learning has shown promise for automated arrhythmia diagnosis, single-model architectures often lack sufficient performance in distinguishing these two arrhythmia types. This study seeks to address the limitations of individual deep learning models and boost classification accuracy for premature beats and atrial fibrillation. It proposes an arrhythmia classification model integrating multiscale feature enhancement and bidirectional temporal dependency. First, a four-layer convolutional residual module with skip connections extracts multiscale local electrocardiogram (ECG) features. Then, multi-head self-attention strengthens critical feature global correlations. Next, a bidirectional long-term temporal de-pendency network captures sequence contextual dependencies. Finally, a Dropout-regularized fully connected layer enables six-type arrhythmia classification. Experiments on a fused dataset (MIT-BIH arrhythmia, MIT-BIH atrial fibrillation, and CODE datasets) yield an overall accuracy of 98.55% and F1-score of 0.9531. Notably, the F1-scores for premature beats (0.9916) and atrial fibrillation (0.9888) outperform recent literature by 2.16% and 4.39%, respectively. The model demonstrates robust classification performance with effective identification of the target arrhythmias, highlighting its potential as a supportive tool for automated ECG diagnosis.

## Simple Summary

Heart rhythm disorders like premature heartbeats and atrial fibrillation pose serious health risks, yet accurate detection remains a key medical challenge. While deep learning tools show promise for automated diagnosis, single computing models often struggle to reliably distinguish these two conditions. This research aimed to address these model weaknesses and boost detection accuracy for the two disorders. We built a combined computing model that captures different levels of heart signal details and tracks rhythm patterns over time: it first extracts fine-grained data from heart monitoring signals via a hierarchical feature extraction structure, highlights key signal connections, and tracks rhythm trends forward and backward in time before sorting rhythms into six categories with error reduction. Tested on three major heart data sets, the model achieved 98.55% overall accuracy, with better performance in identifying premature beats and atrial fibrillation than recent research. It can help doctors diagnose rhythm disorders more reliably, improving care for at-risk patients and advancing public heart health.

## 1. Introduction

Arrhythmia is the main cardiovascular disease that causes sudden cardiac death, of which premature beats and atrial fibrillation are two common types of arrhythmias. They can cause symptoms such as palpitations, chest tightness and dizziness, and may even lead to serious consequences such as heart failure and stroke [1]. Currently, clinical diagnosis is mainly based on electrocardiogram (ECG) analysis [2]. Physicians usually diagnose the disease by recognizing the ECG based on professional knowledge and clinical experience, but manual interpretation is highly subjective and prone to misdiagnosis and omission. In recent years, machine learning has developed rapidly and has been widely used in arrhythmia detection [3]. However, traditional machine learning relies on manual feature extraction, which is time-consuming and laborious [4].

Deep learning, as an efficient machine learning tool [5], has the advantage of being able to learn the feature representation of data autonomously without manual feature extraction [6]. Convolutional Neural Networks (CNNs) [7], with powerful feature extraction capability, have seen successful applications in areas such as speech and image recognition, and are also suitable for ECG feature extraction [8]. Rajpurkar et al. [9] constructed a 34-layer CNN model to achieve arrhythmia detection, and the accuracy on a self-constructed dataset reached 80%. Wang et al. [10] designed an improved 7-layer CNN structure with 99.06% accuracy on the MIT-BIH dataset. However, the traditional single convolution structure has limitations in extracting the dynamic features of long-term ECG signals. This is because CNNs have difficulty fully capturing the temporal dependency and morphological continuity among P waves, QRS complexes, and T waves in ECG signals. Moreover, deep CNNs are also prone to information loss due to gradient degradation, which affects their sensitivity to subtle waveform differences.

Singh et al. [11] used Recurrent Neural Networks (RNNs) [12], Long and Short-term Memory Network (LSTM) [13] and gated recurrent units for arrhythmia detection on the MIT-BIH dataset, and the results showed that LSTM was higher accuracy and more suitable for ECG timing analysis. Li et al. [14] proposed a hybrid CNN-BiLSTM model using CNN to extract ECG morphological features and bidirectional long and short-term memory network (BiLSTM) to capture timing dependencies with an average accuracy of 99.11% on the MIT-BIH dataset. Luo et al. [15] introduced the attention mechanism to construct the CNN-BiLSTM-Attention model and achieved 94.65% accuracy and 98.69% specificity on the MIT-BIH dataset. However, these studies are all based on single-beat segmentation data and only analyzed the morphological characteristics of a single heartbeat. They were unable to model pathological features such as the sporadic nature of premature beats and the long-term rhythm disorder of atrial fibrillation, which require multi-cycle context support. Although the above methods achieved improved performance by introducing BiLSTM and the attention mechanism, respectively, they all adopted the Association for the Advancement of Medical Instrumentation (AAMI) criteria for classification. This standard divides rhythms into five major categories: normal beat (N), supraventricular ectopic beat (S), ventricular ectopic beat (V), fusion beat (F), and unknown beat (Q). Notably, these methods failed to accurately identify and categorize clinically specific arrhythmias, such as premature beats and atrial fibrillation.

Aiming at the above problems, this paper designs the convolutional residual module to solve the accuracy degradation problem caused by information loss and gradient degradation of traditional convolutional layers in deep networks with the help of jump connections. Meanwhile, the combination of Multi-Head Self-Attention Mechanism (MHSA) and BiLSTM overcomes the limitation of a single model to characterize ECG signals by multi-scale enhancement of local and global features. The final MFE-BiLSTM model is constructed with the goal of achieving accurate detection and classification of arrhythmias, including premature beats and atrial fibrillation.

## 2. Materials and Methods

### 2.1. MFE-BiLSTM Model

**Figure 1:**
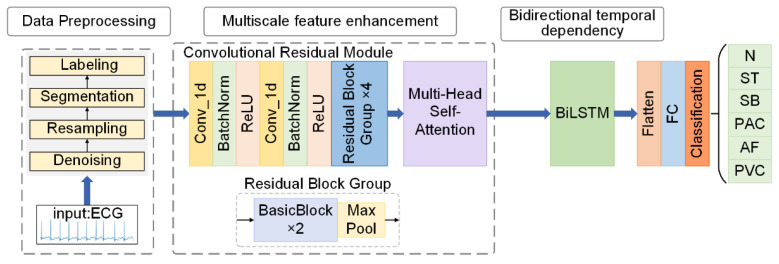
Overall flow of the model. Abbreviations: FC: fully connected layer; N: normal beat; ST: sinus tachycardia; SB: sinus bradycardia; PAC: premature atrial contraction; AF: atrial fibrillation; PVC: premature ventricular contraction.

Figure 1 demonstrates the overall process based on MFE-BiLSTM model. Firstly, the ECG signal is preprocessed and fed into the convolutional residual module to extract the multi-scale local morphological features of the ECG signal. Then, eight parallel attention heads are designed to model the global context of the feature sequence, focusing on enhancing the characterization of the abnormal waveform features. After that, BiLSTM is used to further extract the global bidirectional long-range timing dependencies to establish a complete timing characterization of the ECG signal. Finally, the classification task is implemented by a fully connected layer that includes dimensional compression and dropout regularization.

### 2.2. ECG Dataset and Data Preprocessing

In this paper, MIT-BIH arrhythmia dataset [16], MIT-BIH atrial fibrillation dataset [17], CODE dataset [18] and the China Physiological Signal Challenge (CPSC) 2018 dataset [19] are used for experiments. The details of those datasets are shown in Table 1.

As shown in Table 1, lead II ECG signals are uniformly used for analysis. Data preprocessing includes denoising, resampling, segmentation and labeling to ensure the effectiveness of model training.

Clinical ECG signals are susceptible to industrial frequency interference (50/60 Hz), electromyographic noise and baseline drift [20]. Specifically, we implemented a wavelet-based approach using the Daubechies 6 (Db6) wavelet with 5 decomposition levels. The baseline wander was corrected by fitting and subtracting a third-order polynomial from the approximation coefficients. For noise suppression, a universal threshold was applied using soft-thresholding to the detail coefficients, where σ is the median absolute deviation of the finest detail coefficients divided by 0.6745. The Db6 wavelet’s morphology offers a good balance for analyzing ECG signals, effectively eliminating noise while preserving critical morphological features [14].

(1) $$u_{thresh}=\sigma \times \sqrt{2\log(N)}$$

As shown in Table 1, the sampling rates of the ECG signals in the four datasets are 360 Hz, 250 Hz, 400 Hz and 500 Hz, respectively, and different sampling rates lead to the problems of incompatible input sizes and time-frequency feature distortion. Uniform resampling to 500 Hz standardizes the input size to match the network structure, eliminating the time-domain distortion of the heartbeat waveform caused by different sampling rates and avoids bias in feature recognition.

The ECG signals of the MIT-BIH arrhythmia, MIT-BIH atrial fibrillation, CODE and CPSC 2018 datasets are uniformly segmented into a sequence of 4096 sample points. Segments were extracted by centering a 4096-point window on expert-annotated R-peaks, with a strict non-overlap constraint enforced between consecutive segments. For the problem of insufficient original length of the CODE dataset, the method of zero padding to this length is used for standardization to ensure the consistency of input size of different datasets.

The annotation labels of the four datasets are in the form of letters or symbols, and the symbolic labels are converted to numerical values using the One-Hot Encoding technique. The method maps each category to a binary vector, where only the corresponding position element is 1 and the rest is 0, thus forming the code for each category. It prevents the model from mistaking the coded value magnitude relationship as a feature and focuses only on whether the codes are equal or not [20]. The results of the data labeling are shown in Table 2.

After the above preprocessing, the data of the four public datasets are processed as ECG signal sequences. These sequences have a sampling rate of 500 Hz and a length of 4096 sample points. All sequences contain database-labeled category annotations. The first three dataset sequences are merged to form the single-lead ECG fusion dataset of this paper. The dataset contains 279,914 ECG signal sequences, which can provide reliable data support for model training and testing. The CPSC 2018 dataset is used for verifying the generalization ability of the model and is not involved in the training process of the model. The specific number of heart rhythm types in the fusion dataset is shown in Table 3.

The experiment was conducted in two paradigms: intra-patient and inter-patient. Under the intra-patient paradigm, the dataset was randomly divided in a ratio of 8:1:1, as shown in Table 4.

Under the inter-patient paradigm, all ECG records are divided into training sets and test sets according to patients to ensure that the ECG records of the same patient will not be leaked across datasets, as shown in Table 5.

The number of labels of different heart rhythm types in the training set and test set under the inter-patient paradigm is shown in Table 6.

### 2.3. Multiscale Feature Enhancement

#### 2.3.1. Hierarchical Feature Extraction

The module builds a hierarchical feature extraction architecture using convolutional layers and multi-level residual block groups. This design enables multi-scale feature extraction of ECG signals, spanning from micro-level waveform details to macro-level rhythm patterns. This addresses the issue of accuracy decline in traditional convolutional layers in deep networks due to information loss and gradient degradation, enabling the model to efficiently learn complex feature mappings. ECG signals are one-dimensional time series, so one-dimensional convolutional layers are used to extract ECG signal features [21]. The convolutional layer uses large time window convolution kernels to capture morphological features such as QRS complexes and T waves, and reduces computational complexity through down sampling with a stride of 2, while retaining the global information of the ECG rhythm.

**Figure 2:**
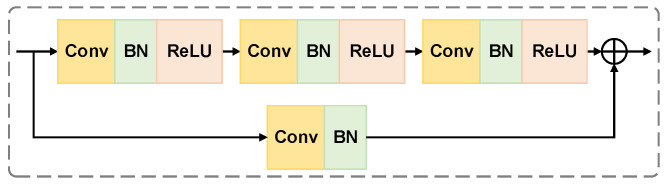
BasicBlock structure. Abbreviations: Conv: Convolution; BN: Batch Normalization; ReLU: Rectified Linear Unit. Color definition: yellow blocks denote Conv layers, light green blocks denote BN layers, and light orange blocks denote ReLU activation functions. Solid arrows indicate data flow direction. The block includes two paths: the top path consists of three sequential “Conv-BN-ReLU” standard cell structures; The bottom path is a residual connection. The circled symbol represents element-level summation of outputs from the top main path and the bottom residual path.

The residual block group adopts a pyramidal hierarchical structure with the number of output channels doubling at each layer (32-64-128-256), and the core is the BasicBlock. The specific structural design is shown in Figure 2. This block contains three sequentially connected one-dimensional convolutional layers. Each layer is followed by Batch Normalization (BN) and Rectified Linear Unit (ReLU) activation functions. They form a standard cell structure of “Conv-BN-ReLU”. The residual connection employes 1 × 1 convolution with batch normalization for dimension matching. This enables element-level summation between the main path output and the constant mapping feature. Such design effectively alleviates the gradient vanishing problem. This design gradually abstracts the low-level waveform features into high-level diagnostic features, and finally outputs a multi-scale feature representation, providing a powerful and robust feature basis for the ECG classification task.

#### 2.3.2. Multi-Head Self-Attention

Abnormalities in ECG signals (such as atrial fibrillation) may manifest as combined abnormalities of multiple discontinuous bands. The multi-head self-attention mechanism performs parallel computing across multiple attention sets. It captures feature relationships at different positions of the input sequence through distinct subspaces, enabling simultaneous focus on scattered abnormal features. This design enhances the model’s ability to perceive long-distance temporal dependencies in ECG signals. To balance the computational efficiency and feature expression ability, 8 independent and parallel attention heads are selected. The input feature dimension is 256, which matches the number of output channels of the residual block group. The single head dimension is 32, decomposing high-dimensional computations into multiple low-dimensional computations to reduce the number of parameters and computational complexity.

**Figure 3:**
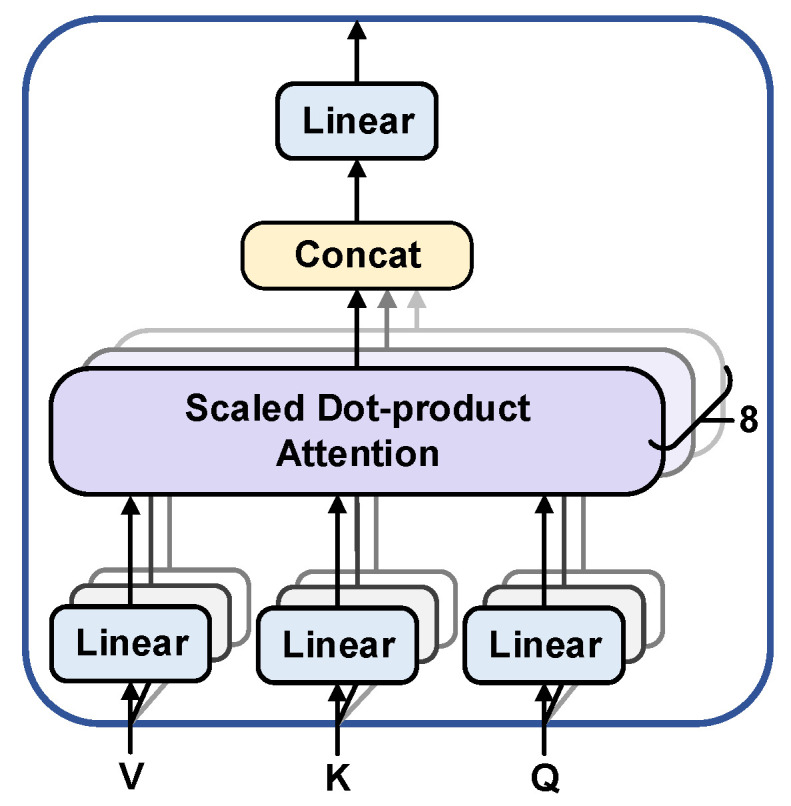
Multi-head self-attention mechanism structure. Abbreviations: Q: Query vector; K: Key vector; V: Value vector. Color illustration: distinct background colors mark different functional modules: light blue for Linear layers, beige for the Concat module, and light purple for the Scaled Dot-product Attention module. Solid arrows indicate the direction of data flow.

Each attention head separately performs a learnable linear projection of the input sequence to generate Query, Key and Value vectors. Dynamic association weights between any two time points are computed by scaled dot product attention. These weights capture multiscale dependencies spanning local beats to global rhythms. The outputs from the final eight attention heads are fused through concatenation. A linear transformation is then applied to produce an enhanced feature representation. This representation integrates global contextual information. While a single attention head may focus only on specific patterns, multiple heads capture multi-scale features in parallel by splitting the input vectors into different subspaces [22]. The specific structure is shown in Figure 3.

### 2.4. Bidirectional Temporal Dependency

In the ECG classification task, BiLSTM can effectively model the waveform temporal characteristics in the heartbeat cycle (such as the anterior and posterior correlations of P waves, QRS groups, and T waves), improve the recognition ability of abnormal ECG waveforms, and solve the problem of insufficient modeling of complex rhythms by unidirectional models. LSTM dynamically regulates the storage and forgetting of temporal information by introducing cell states and gating mechanisms. By optimizing the gradient flow, not only the long-term dependence of the sequence is retained, but also the gradient vanishing and explosion problems of the traditional RNN are effectively alleviated [23].

**Figure 4:**
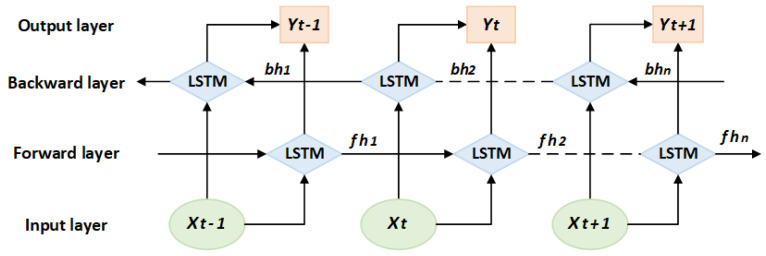
BiLSTM structure. Abbreviations: LSTM: Long Short-Term Memory; *X*: input feature; Y: output of the BiLSTM module; *fh*: forward hidden state; *bh*: backward hidden state. Color illustration: light green circles represent the Input layer; light orange rectangles represent the Output layer; light blue diamonds represent LSTM units. Solid arrows indicate data flow direction: the Forward layer transmits data in the forward time direction, while the Backward layer transmits data in the backward time direction.

The BiLSTM module adopts a bidirectional structure design, with the input being 256-dimensional features captured by the multi-head self-attention module. The forward and backward long-term dependencies of ECG signals are synchronously captured through bidirectional timing modeling. By concatenating the forward and backward hidden states, the output dimension is expanded to 512, enhancing the feature expression ability. The network structure of BiLSTM is shown in Figure 4.

Here, $X_{t}$ is the input feature at time t, the forward hidden vectors $fh_{1}\sim fh_{n}$ capture the historical timing information, and the backward hidden vectors $bh_{1}\sim bh_{n}$ extract the future timing correlation. After BiLSTM synchronously parses the forward and backward timing features, the bidirectional hidden vectors at time t are spliced together to form the output $Y_{t}$, which integrates the contextual information.

The convolutional layer and residual block groups extract local waveform features, the multi-head self-attention module models the global dependency relationship, and BiLSTM further captures the temporal dynamic features to form a hierarchical feature extraction process. The BiLSTM output is randomly inactivated by the Dropout layer to alleviate overfitting, and then feature dimension reduction and classification are completed through the fully connected layer. Finally, six types of ECG classification results are output.

The MFE-BiLSTM model is constructed based on the above modules, and the design parameters of this model are shown in Table 7.

### 2.5. Experimental Configuration and Evaluation Indicators

The hardware configuration of the experimental platform is Intel i5-11400H CPU, NVIDIA RTX 3050 graphics card, 16G running memory, and the model is based on python 3.7.12 and PyTorch 1.10.1 framework. The key training hyperparameters were configured as follows: the Adam optimizer was adopted for parameter optimization with an initial learning rate of 0.001; the batch size was set to 128, and the total number of training epochs was 50.

The experiment uses Precision, Recall, Accuracy and F1-score as evaluation metrics, defined as follows. The precision rate represents the ratio of samples that are actually positive cases out of those predicted to be positive by the model, and is used as a measure of how accurate the predictions are.

(2) $$\mathrm{Precision}=\frac{\mathrm{TP}}{\mathrm{TP}+\mathrm{FP}}$$

Recall represents the ratio of the total number of positive case samples correctly predicted by the model to the total number of actual positive case samples, which can intuitively evaluate whether there is any missing classification in the model.

(3) $$\mathrm{Recall}=\frac{\mathrm{TP}}{\mathrm{TP}+\mathrm{FN}}$$

Accuracy represents the ratio of the number of samples correctly predicted by the model to the total number of actual samples, reflecting the performance of the model in the overall classification task.

(4) $$Accuracy=\frac{TP+TN}{TP+TN+FP+FN}$$

The F1-score represents the reconciled average of precision and recall. This combined metric considers the balance between these two measures. It aims to comprehensively evaluate the effectiveness of the model for positive sample classification and to avoid biased evaluations caused by relying only on precision or recall.

(5) $$F1=2\frac{Precision\cdot Recall}{Precision+Recall}$$

Above TP (True Positive) indicates that the true result is true and the predicted result is also true; FN (False Negative) indicates that the true result is true and the predicted result is false; FP (False Positive) indicates that the true result is false and the predicted result is true; TN (True Negative) indicates that the true result is false and the predicted result are also false [24].

## 3. Results

### 3.1. Test Experiment Results

**Figure 5:**
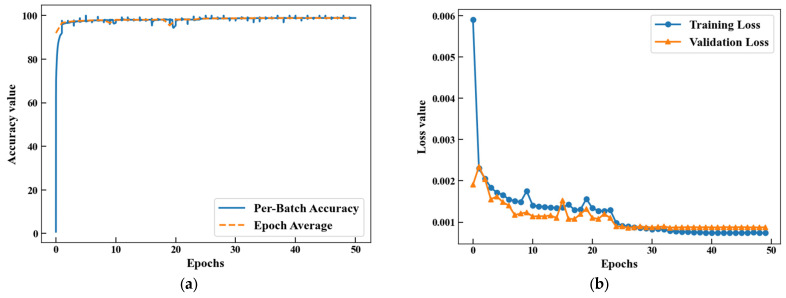
(**a**) Training set accuracy curve. (**b**) Loss function curve.

The experimental dataset comprises 279,914 preprocessed single-lead ECG signal sequences. The fusion dataset is divided into training, validation and test sets according to the ratio of 8:1:1. The resulting subset sizes are 223,931, 27,991, and 27,992 sequences, respectively. Training set accuracy and loss function curves are shown in Figure 5. During training, the accuracy rate shows an increasing trend, and the rate of increase gradually slows down until it eventually stabilizes. Conversely, the loss function curve demonstrates a gradually decreasing trend, and eventually also reaches a stable state and stays at a low level.

**Figure 6:**
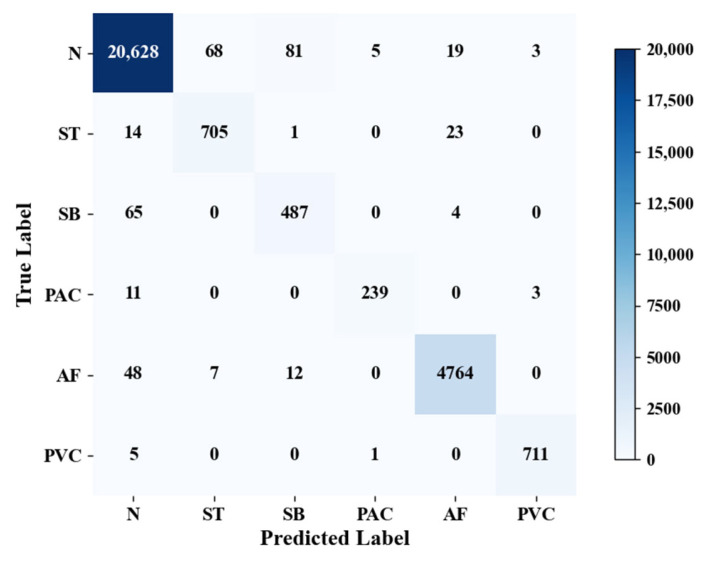
Confusion matrix of test results. The color intensity in the matrix corresponds to the number of samples for each true-predicted label pair: darker shades (closer to dark blue) indicate a larger sample count, while lighter shades (closer to white) indicate a smaller sample count. The color bar on the right displays the specific sample count range matching each color shade.

The experiment uses a split test set to verify the performance of the model, and the confusion matrix is shown in Figure 6, where the rows represent the predictions of the model and the columns represent the true labels. As shown in Table 8, the overall accuracy and F1-score of the model on the test set are 98.55% and 0.9531, respectively.

The receiver operating characteristic (ROC) curve and the area under the curve (AUC) are used to evaluate the classification performance of the model. The ROC curve is based on the False Positive Rate (FPR) as the horizontal axis and the True Positive Rate (TPR) as the vertical axis, visualizing the distribution of classification performance [25]. AUC quantifies the model performance by calculating the percentage of the area under the curve (normalizing the area of the entire coordinate space to 1), with higher values indicating better classification results. The calculation formula is shown in Equations (6) and (7).

(6) $$FPR=\frac{FP}{FP+TN}$$

(7) $$TRP=\frac{TP}{TP+FN}$$

Macro-AUC reflects the average discriminatory power of the model in each category, while Micro-AUC focuses on the global impact of dominant categories. The former calculates the mean AUC of each category equally, while the latter is weighted by the sample size, and the combination of the two can comprehensively analyze the classification effectiveness of the model. The formulas are shown in Equations (8) and (9).

(8) $$\text{Macro-AUC}=\frac{1}{C}\sum_{i=1}^{c}{AUC}_{i}$$

where ${AUC}_{i}$ is the AUC value of category i as a positive category. A single AUC is calculated after constructing a global confusion matrix by merging the predicted probabilities and true labels of all classes.

(9) $$\text{Micro-AUC}=\mathrm{AUC}\left(\frac{\sum {TP}_{i}}{\sum \left({TP}_{i}+{FN}_{i}\right)},\frac{\sum {FP}_{i}}{\sum \left({FP}_{i}+{TN}_{i}\right)}\right)$$

**Figure 7:**
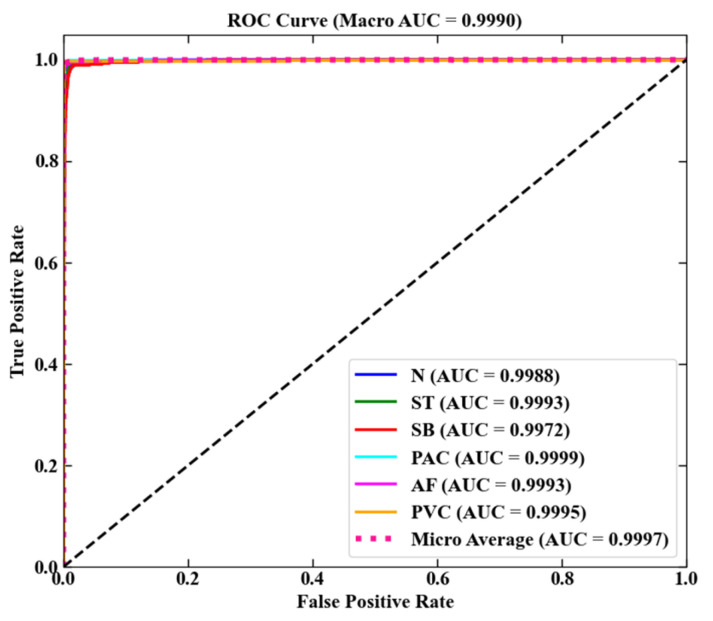
ROC curve. The curves exhibit partial overlap because the AUC values of all categories (and averages) are extremely high and closely clustered (ranging from 0.9972 to 0.9999). To ensure readability, the specific AUC value for each category is labeled directly in the legend, enabling clear distinction of performance across classes.

The ROC curves of the model on the test set are shown in Figure 7, and the experimental test results are shown in Table 8. The Macro-AUC and Micro-AUC of the model on the test set reach 0.9990 and 0.9997, respectively, and the ROC curve distributions of different rhythm types are almost the same, indicating that the model has the effective ability to detect the six types of heart rhythms.

### 3.2. The Generalization Verification Experiment Results

ECG signals exhibit individual specificity. If the same patient’s data appears simultaneously in both the training set and the test set, it will lead to overly optimistic model evaluation results, which cannot truly reflect its generalization ability on new patients. Therefore, the data are divided into two paradigms: intra-patient and inter-patient. Under the inter-patient paradigm, a strict patient-independent partitioning strategy is adopted to ensure that data from the same patient only appears in the training set or the test set, avoiding the risk of data leakage. The model performance results under the inter-patient paradigm are shown in Table 9.

**Figure 8:**
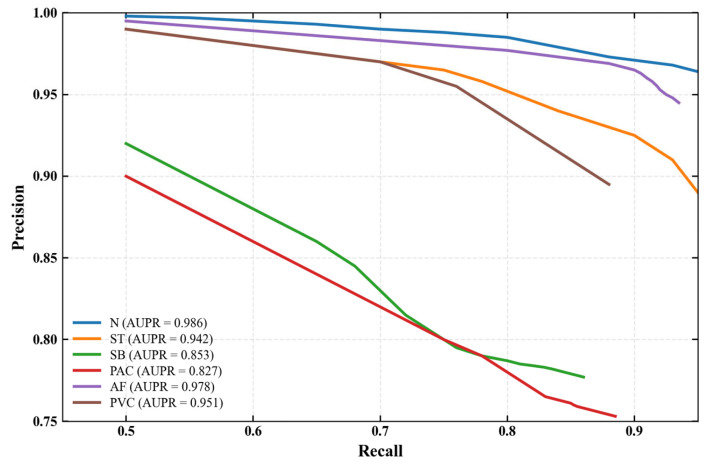
Precision-Recall curves of arrhythmia classification under inter-patient paradigm.

To better characterize the performance of each arrhythmia class—especially the rare PAC and low-performance SB—and visualize the trade-off between precision and recall, we supplement precision-recall (PR) curves for all six rhythm categories and calculate the area under the PR curve (AUPR) as a quantitative metric, as shown in Figure 8. Compared with ROC curves, PR curves are more sensitive to class imbalance and rare events, making them a more robust evaluation tool for datasets with uneven sample distributions [26].

Under the inter-patient paradigm, the performance indicators of the model have decreased to a certain extent compared with those under the intra-patient paradigm in Table 8, with relatively low performance in the SB and PAC categories—this is further validated by the supplementary PR curves and AUPR metrics. As shown in Table 9 and Figure 8, SB (F1 = 0.8057, AUPR = 0.853) features slow heart rate and high morphological similarity to N, and its PR curve exhibits a moderate precision decline with increasing recall. This makes it prone to misjudgment, especially as individual variability in cardiac electrical activity under the inter-patient setting blurs the boundary between SB and N. For PAC (F1 = 0.8762, AUPR = 0.827), the number of samples is small (Table 6) and ECG varies significantly across patients. Its PR curve shows a steeper precision decline, a typical characteristic of rare classes, leading to relatively low recognition accuracy. The AUPR values further quantify these challenges: SB and PAC have the lowest AUPR among all classes, confirming that their reduced performance stems from morphological similarity and sample scarcity.

To further verify the generalization of the model, it was tested on the CPSC 2018 dataset. Since this dataset does not include all six heart rhythm types classified by the model, four identical types were selected for testing. The specific results are shown in Table 10.

As shown in Table 10, the performance of the model is affected on untrained datasets. Among them, the performance of PAC and AF categories is relatively low. The number of samples in the PAC category is small, and the differences among patients in different datasets of the AF category are significant, resulting in low classification performance.

### 3.3. Comparison Experiment Results

Based on the fusion dataset, the ablation experiments are conducted by using CNN, CNN-BILSTM, CNN-MHSA and the MFE-BiLSTM model. The test results are shown in Table 11.

The ablation experiments show that both the multi-head self-attention module and BiLSTM have an improving effect on the classification performance of the model, indicating the key role of global feature association in anomaly location and the necessity of bidirectional time series modeling for rhythm analysis. This study combines multi-scale feature enhancement with bidirectional time-dependent modeling to form a three-level processing framework of “local feature extraction, global correlation enhancement, and time-series dynamic modeling”, reducing the one-sided influence of the traditional single model on the characterization of ECG signals.

**Figure 9:**
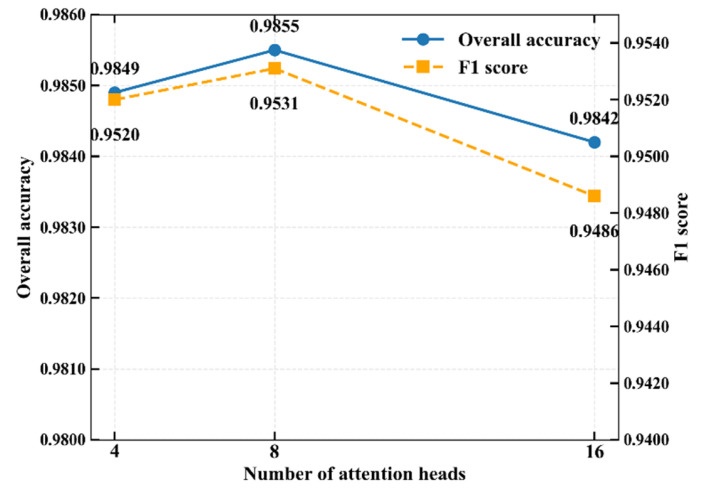
Comparison of results of different attention head designs.

To verify the balance between computational efficiency and feature capture capability of the 8-head attention mechanism, a comparative experiment with different attention head configurations was conducted. As shown in Figure 9, the 8-head configuration model has better classification performance and is superior to the 4-head and 16-head schemes. Considering the overall computational efficiency, the 8-head attention mechanism design was ultimately selected for the model.

To verify the effectiveness of the hierarchical design of multi-scale feature enhancement in the model, while keeping all other modules fixed, a comparative experiment was conducted solely on the multi-scale feature enhancement module. A comparative experiment was conducted between a model with a complete multi-scale module and one that only uses a single-scale convolution kernel module. The experimental results are shown in Table 12.

As can be seen from Table 12, with the increase in single-scale convolution kernels, the classification performance of the model gradually improves, but the number of model parameters also gradually increases. The multi-scale module designed in the article has better performance compared with the single-scale module. Meanwhile, the number of model parameters is controlled, indicating the effectiveness of the multi-scale hierarchical design.

The model was compared with methods used in the literature in recent years to identify arrhythmia types including premature beats and atrial fibrillation from ECG signals. A comparison of the model results is shown in Table 13.

As shown in Table 13, the model achieves the highest F1-score on five of the six rhythm types (N, ST, PAC, AF, PVC), with the F1-scores of PAC, PVC and AF reaching 0.9621, 0.9916 and 0.9888, respectively. It should be noted that there are inevitable heterogeneities in datasets and evaluation metrics among the compared methods, which may introduce potential confounding factors for direct performance comparison. From the perspective of dataset differences: (1) Data sources vary across studies, including public benchmarks (e.g., MIT-BIH, CPSC 2018) and private clinical datasets (e.g., clinical ECG data from the First Affiliated Hospital of Zhejiang University School of Medicine), leading to discrepancies in case distribution, arrhythmia prevalence, and signal noise levels; (2) Data duration and scale differ significantly—for instance, some methods use short-segment data (6–45 s) that may lack long-term rhythm dynamic information, while others adopt 30-min or 10-h long-segment data that contain more comprehensive temporal features but may increase interference from motion artifacts or baseline drift. From the perspective of evaluation metric differences: (1) Partial methods (e.g., Wang et al. [27], Murugesan B et al. [29]) do not report F1-scores for ST and SB rhythms, resulting in incomplete coverage of the target arrhythmia types; (2) Individual studies only provide overall accuracy without detailed per-class F1-scores, making it difficult to assess performance on specific arrhythmia subtypes. These inconsistencies may lead to overestimation or underestimation of certain methods’ performance: short-segment datasets may favor models sensitive to local morphological features but fail to reflect performance on long-term rhythm disorders, while private datasets with unreported distribution characteristics may limit the generalizability of their results.

This result indicates that the collaborative modeling of multi-scale feature enhancement and bidirectional temporal dependence can effectively capture the association between local waveform abnormalities (such as widened QRS waves) and global rhythm disorders (such as irregular RR intervals) in ECG signals, reducing the limited impact of a single model on the characterization of complex arrhythmias. The high F1-score (0.9888) of atrial fibrillation classification indicates that the model accurately captures the global distribution characteristics of irregular f waves in atrial fibrillation signals through the multi-head self-attention mechanism, while BiLSTM strengthens the dynamic modeling of the temporal fluctuations of f waves before and after. Notably, despite these heterogeneities, our model still delivers superior per-class and overall performance. It was validated across multiple public datasets (MIT-BIH series, CPSC 2018, CODE) with diverse durations (7 s–10 h) and comprehensive evaluation metrics (per-class F1-score, overall accuracy, overall F1-score). This confirms two key points: first, the framework’s ability to integrate local and global features is robust to dataset variations; second, its performance advantages do not rely on specific data characteristics or incomplete evaluation metrics. The experimental results demonstrate superior performance in arrhythmia classification tasks, particularly exceling at detecting premature beats and atrial fibrillation.

## 4. Discussion

The MFE-BiLSTM model constructed in this study achieved an overall accuracy rate of 98.55% and an F1-score of 0.9531 on the fused dataset. This achievement not only validates the effectiveness of collaborative modeling integrating multi-scale feature enhancement and bidirectional temporal dependency learning but also provides a novel theoretical reference for feature modeling in intelligent arrhythmia diagnosis.

From the perspective of feature extraction mechanism, the effectiveness of the MFE-BiLSTM model stems from the hierarchical design of the local-global-temporal three-level feature processing framework, which is highly consistent with the classical theories of deep learning in temporal medical signal analysis. In this study, the effectiveness of hierarchical feature extraction was validated through the design of multi-scale convolutional kernels. Compared with single-scale convolutional kernels, the multi-scale module achieved higher classification accuracy under comparable parameter quantity control, indicating that it can better balance local details and global contextual information.

More importantly, this framework possesses a profound physiological significance that aligns with clinical diagnostic logic. The multi-scale convolutional module is not merely a technical design but reflects the multi-resolution analysis inherent in electrocardiography. The smaller-scale kernels excel at capturing fine-grained, localized morphological features. These include the precise shape and duration of the P wave, QRS complex, and T wave. This process is analogous to a cardiologist zooming in to examine specific waveform deflections during clinical interpretation. Conversely, the larger-scale kernels capture broader contextual information, such as the overall trend of the ST segment or the baseline between beats, which is crucial for diagnosing conditions like ischemia. The residual connections ensure the seamless integration of these multi-level features, preventing the loss of critical fine details while incorporating broader context, mirroring the diagnostic process of correlating local abnormalities with the overall rhythm strip. This design draws on the residual idea of ResNet [28]: skip connections are employed to alleviate the gradient degradation problem in deep networks, while pyramid-style channel expansion is adopted to enhance feature diversity.

The core idea of the attention mechanism proposed by Vaswani et al. [22] lies in focusing on key features through dynamic weight assignment. In this study, the 8-head self-attention module is precisely based on this theory, which is adapted to the long-range rhythm analysis of ECG signals. Compared with a single attention head, multi-head subspace division can simultaneously capture the scattered f-wave features in AF signals and the morphological abnormalities of QRS complexes in premature beat signals. This reflects the core value of the attention mechanism in accurately locating abnormal features within medical time-series signals, which echoes the findings of Zhou et al. [31] in the frequency-domain convolutional attention mechanism.

The multi-head self-attention mechanism offers a powerful computational analogy for analyzing complex rhythm disorders like atrial fibrillation (AF). The scattered and inconsistent f-waves in AF present a significant challenge, as they vary in amplitude, frequency, and morphology. The multi-head attention mechanism effectively addresses this by allowing the model to project the input signal into multiple subspaces. In this way, different attention “heads” can learn to focus on different aspects of the f-waves simultaneously. One head might attend to regions with high-frequency oscillations, another to subtle low-amplitude fibrillatory activity, and yet another to the irregular RR intervals caused by AF. This parallel processing capability enables the model to comprehensively aggregate the dispersed signatures of AF, significantly enhancing its detection accuracy. This process is conceptually similar to an expert scrutinizing different segments of a long-term ECG recording to confirm the presence of AF. The introduction of the BiLSTM module follows the bidirectional recurrent structure theory proposed by Greff et al. [23]. By modeling temporal information in both forward and backward directions, it enhances the ability to recognize rhythm types (such as ST and SB) that rely on the continuity of RR intervals. This validates the necessity of bidirectional temporal dependency for modeling the dynamic features of cardiac rhythms.

From a clinical application perspective, this study focuses on the accurate classification of clinically high-priority arrhythmias such as premature beats and AF, addressing the limitation of traditional models classified according to the AAMI standard that prioritize category coverage over clinical relevance. Studies by Murugesan et al. [29] and Huang et al. [30] have both pointed out that existing arrhythmia classification models mostly focus on general category division, with insufficient recognition accuracy for specific conditions such as premature beats and AF. In this study, the multi-scale convolutional residual module preserves the microcosmic waveform details of ECG signals, and combined with the global correlation modeling of multi-head self-attention, high-precision recognition of premature beats and AF is achieved. The model’s capability to reliably identify these clinically critical arrhythmias from single-lead ECG signals makes it an ideal candidate for automated ECG analysis software in telemedicine platforms. This could greatly facilitate early detection and prolonged screening of paroxysmal AF, which is often asymptomatic and prone to be missed in routine short-term examinations. For primary care physicians or clinicians in resource-limited settings, such an automated tool can serve as a valuable decision-support system, flagging potential high-risk arrhythmias for further expert review, thereby improving diagnostic efficiency and reducing the rate of missed diagnoses.

Notably, the MFE-BiLSTM model’s compact architecture confers inherent advantages for integration into wearable ECG devices, which are typically constrained by limited computing power, battery life, and hardware size. Compared with larger models, our model balances performance and computational efficiency, which is a key prerequisite for wearable deployment. While the current study focuses on classification accuracy and generalization, we recognize that inference latency and real-time capability are critical for practical clinical application in wearables. To address this, future work will: (1) Quantify the model’s floating-point operations (FLOPs) to characterize computational complexity; (2) Measure inference time per 4096-sample ECG segment on low-power hardware platforms representative of wearable device specifications; (3) Optimize the model via quantization or pruning techniques to further reduce latency without compromising accuracy. These steps will provide direct evidence of the model’s real-time feasibility for wearable ECG systems.

However, this study still has certain limitations. Firstly, the model exhibits relatively low performance in the SB category, with over 90% of misclassifications being incorrectly judged as the N category. This is closely related to the characteristics of SB rhythm: although it presents with bradycardia, its waveform morphology is highly similar to that of normal rhythm, making it difficult for the model to distinguish them based solely on morphological features. Notably, SB is essentially different from N in temporal rhythm characteristics: SB shows abnormally prolonged RR intervals, significantly reduced heart rate variability (HRV) indices, and poor regularity of RR interval sequences, whereas N maintains stable RR intervals and normal HRV levels, which constitute the core discriminative basis between the two rhythms. This phenomenon is consistent with the bradycardia misclassification problem observed by Murugesan et al. [29] on the MIT-BIH dataset. Future research can introduce a dedicated rhythm feature extraction branch for auxiliary judgment, which focuses on quantitatively analyzing the RR interval sequence variability features and typical HRV metrics of cardiac signals, and then performs joint discrimination by synergistically combining waveform morphological features and rhythm dynamic patterns. This integrated feature learning strategy is expected to effectively overcome the limitation of single morphological feature-based recognition and substantially improve the classification accuracy of SB rhythm. Secondly, this study only used single-lead (lead II) ECG data without fusing multi-lead information, which may result in the loss of critical spatial dimensional diagnostic information reflecting the cardiac electrical activity distribution across different cardiac regions. Huang et al. [30] pointed out that multi-lead ECG signals have prominent complementary advantages in identifying cardiac arrhythmias by providing multi-angle spatial feature cues, which is particularly valuable for improving the diagnostic accuracy of complex rhythm abnormalities. In the future, we will prioritize incorporating multi-lead ECG data represented by precordial leads (V1-V6) and construct a cross-lead attention mechanism based on the multi-lead signals. This mechanism will automatically capture the interdependent correlation and spatial feature complementarity among different leads, adaptively assign attention weights to discriminative lead features, and realize deep fusion of single-lead morphological features and multi-lead spatial features. This optimized technical framework is expected to fully compensate for the spatial information loss caused by single-lead data input, and further enhance the generalization ability and clinical practicality of the model in real-world complex diagnostic scenarios.

## 5. Conclusions

To address the pressing clinical need for accurate and automated detection of high-risk arrhythmias, this study developed the MFE-BiLSTM model, which synergistically integrates multi-scale feature enhancement with bidirectional temporal dependency learning. The model demonstrated superior performance, particularly in the critical tasks of classifying premature beats and atrial fibrillation, which are central to stroke and sudden cardiac death risk assessment. The key innovation lies in the model’s hierarchical architecture, which mirrors clinical reasoning: the multi-scale convolutional residual module meticulously captures localized waveform abnormalities, while the multi-head self-attention mechanism globally identifies scattered signatures of AF. The BiLSTM network then contextualizes these findings within the rhythm’s temporal evolution.

The primary clinical value of this research is its potential to be deployed as a robust decision-support tool. By enabling high-accuracy, automated analysis of ECG signals, the model can significantly aid in screening programs, enhance the diagnostic yield of long-term ambulatory monitoring, and streamline the workflow in busy clinical settings. This contributes directly to the goal of timely intervention and improved patient outcomes. Future work will focus on incorporating multi-lead information and rhythm-based features to further enhance its clinical applicability and generalizability across diverse patient populations.

## Figures and Tables

**Table 1:** ECG dataset.

Dataset	MIT-BIH Arrhythmia Dataset	MIT-BIH Atrial Fibrillation Dataset	CODE Dataset	CPSC 2018
Source	Boston’s Beth Israel Hospital	Boston’s Beth Israel Hospital	Telehealth Network of Minas Gerais	Southeast University and 11 hospitals
Number of records	48	25 (records 00735 and 03665 are not available without data files)	18	6877
Record duration	30 min	10 h	7–10 s	6–60 s
Sample frequency/Hz	360	250	400	500
Number of leads	2	2	12	12

**Table 2:** Data labeling.

Types of Arrhythmias	One-Hot Encoding
N (normal beat)	1, 0, 0, 0, 0, 0
ST (sinus tachycardia)	0, 1, 0, 0, 0, 0
SB (sinus bradycardia)	0, 0, 1, 0, 0, 0
PAC (premature atrial contraction)	0, 0, 0, 1, 0, 0
AF (atrial fibrillation)	0, 0, 0, 0, 1, 0
PVC (premature ventricular contraction)	0, 0, 0, 0, 0, 1

**Table 3:** Number of arrhythmia types in the dataset.

	MIT-BIH Arrhythmia Dataset	MIT-BIH Atrial Fibrillation Dataset	CODE Dataset	Total
N	74,700	0	134,657	209,357
ST	0	0	7584	7584
SB	0	0	5605	5605
PAC	2532	0	0	2532
AF	0	40,899	6831	47,730
PVC	7106	0	0	7106
Total	84,338	40,899	154,677	279,914

**Table 4:** The number of labels in the intra-patient paradigm.

Types of Arrhythmias	Training Set	Validation Set	Test Set
N	167,408	21,075	20,874
ST	6048	792	744
SB	4535	513	557
PAC	2039	240	253
AF	38,209	4677	4844
PVC	5692	694	720
Total	223,931	27,991	27,992

**Table 5:** Dataset division under the inter-patient paradigm.

Dataset		ECG Record Number
MIT-BIH arrhythmia dataset	Training set	101 106 108 109 112 114 115 116 118 119 122 124 201 203 205 207 208 209 215 220 223 230
	Test set	100 103 105 111 113 117 121 123 200 202 210 212 213 214 219 221 222 228 231 232 233 234
MIT-BIH atrial fibrillation dataset	Training set	04015 04048 04746 04936 05121 06426 06995 07859 07910 08219 08405 08455
	Test set	04043 04126 04908 05091 05261 06453 07162 07879 08215 08378 08434
CODE dataset	Training set	0 2 4 6 8 10 12 14 16
	Test set	1 3 5 7 9 11 13 15 17

**Table 6:** The number of labels under the inter-patient paradigm.

Types of Arrhythmias	Training Set	Test Set	Total
N	108,419	100,938	209,357
ST	3950	3634	7584
SB	2925	2680	5605
PAC	800	1732	2532
AF	31,014	16,716	47,730
PVC	3899	3207	7106
Total	151,007	128,907	279,914

**Table 7:** Model design parameters.

Layer/Operation	Output Size	Explanation
Input	(B, 1, L)	Input signal, B is the batch size, L is the original sequence length (divisible by 64, such as 4096)
Convolutional layer 1 (k = 60, s = 2)	(B, 32, L/2)	Convolution kernel 60, step 2, padding 29, output length L/2
Convolutional layer 2 (k = 20, s = 2)	(B, 32, L/4)	Convolution kernel 20, step 2, padding 9, output length L/4
Residual block group 1	(B, 32, L/8)	Two residual blocks (hold channel 32), length L/8 after max pooling (s = 2)
Residual block group 2	(B, 64, L/16)	Channels 32–64 with maximum pooled length of L/16
Residual block group 3	(B, 128, L/32)	Channels 64–128 with maximum pooled length of L/32
Residual block group 4	(B, 256, L/64)	Channels 128–256 with maximum pooled length of L/64
Multi-head self-attention	(B, 256, L/64)	Input reshapes to (B, L/64, 256), output restores original shape
BiLSTM	(B, 512, L/64)	Bidirectional output concatenation, feature dimension 512
Flatten	(B, 512 × (L/64))	If L = 4096, it is 512 × 64 = 32,768 after flattening.
Fully connected layer	(B, 128)	Downscaling to 128, applying ReLU and Dropout
Classification layer	(B, 6)	Output 6 classification results

**Table 8:** Arrhythmia classification results under the intra-patient paradigm.

Types of Arrhythmia	Precision	Recall	F1	AUC
N	0.9943	0.9907	0.9925	0.9988
ST	0.8973	0.9529	0.9243	0.9993
SB	0.8365	0.8831	0.8591	0.9972
PAC	0.9718	0.9526	0.9621	0.9999
AF	0.9917	0.9860	0.9888	0.9993
PVC	0.9916	0.9916	0.9916	0.9995
Overall accuracy	0.9855			
Overall F1-score	0.9531			
Macro-AUC	0.9990			
Micro-AUC	0.9997			

**Table 9:** Arrhythmia classification results under the inter-patient paradigm.

Types of Arrhythmias	Precision	Recall	F1	AUC
N	0.9699	0.9349	0.9521	0.9702
ST	0.8992	0.9353	0.9169	0.9972
SB	0.7840	0.8287	0.8057	0.9928
PAC	0.7614	0.8523	0.8762	0.9616
AF	0.9023	0.9142	0.9082	0.9931
PVC	0.9136	0.7746	0.8383	0.9848
Overall accuracy	0.8985			
Overall F1-scores	0.8829			
Macro-AUC	0.9833			
Micro-AUC	0.9853			

**Table 10:** The test results of the model on the 2018 Physiological Signal Challenge dataset.

Types of Arrhythmias	Precision	Recall	F1	Number of Records
N	0.9625	0.9247	0.9432	918
PAC	0.8768	0.8067	0.8403	574
AF	0.7879	0.9510	0.8618	1098
PVC	0.9375	0.8356	0.8836	653
Overall accuracy	0.8912			
Overall F1-score	0.8822			

**Table 11:** Results of ablation experiments.

Model	Accuracy	F1	Macro-AUC	Micro-AUC
CNN	0.9059	0.9087	0.9689	0.9945
CNN-BiLSTM	0.9569	0.9235	0.9865	0.9956
CNN-MHSA	0.9673	0.9212	0.9861	0.9978
MFE-BiLSTM	0.9855	0.9531	0.9990	0.9998

**Table 12:** Comparison results of convolution kernels of different scales.

Model Settings	Precision	F1	Model Parameter Quantity/M
Single-scale convolution kernel: 3	0.9841	0.9503	5.1
Single-scale convolution kernel: 5	0.9843	0.9523	5.4
Single-scale convolution kernel: 9	0.9848	0.9529	5.8
Complete multi-scale module	0.9855	0.9531	5.3

**Table 13:** Comparison of model results.

Literature		Wang et al. [27]	Ye et al. [28]	Murugesan B et al. [29]	Huang et al. [30]	Zhou et al. [31]	Jin et al. [32]	This Article
Model		Unet-LSTM-Attention	ResNet-BiLSTM	CNN-LSTM	multi-feature fusion CNN	CNN-FCBA (frequency convolutional block attention)	CNN-BiLSTM-Attention	MFE-BiLSTM
Training set		CPSC 2018	The 2017 PhysioNet/CinC Challenge, Clinical ECG data of the First Affiliated Hospital of Zhejiang University School of Medicine	MIT-BIH arrhythmia dataset	MIT-BIH arrhythmia dataset	MIT-BIH arrhythmia dataset	The large-scale Chinese ECG dataset jointly constructed by Shanghai First People’s Hospital	MIT-BIH arrhythmia dataset, MIT-BIH atrial fibrillation dataset, CODE dataset
Test set		CPSC 2018	The 2017 PhysioNet/CinC Challenge, Clinical ECG data of the First Affiliated Hospital of Zhejiang University School of Medicine	MIT-BIH arrhythmia dataset	MIT-BIH arrhythmia dataset	MIT-BIH arrhythmia dataset	The large-scale Chinese ECG dataset jointly constructed by Shanghai First People’s Hospital	MIT-BIH arrhythmia dataset, MIT-BIH atrial fibrillation dataset, CODE dataset, CPSC 2018
Scale of data		6877 pieces of data ranging from 6 to 60 s	8528 pieces of data ranging from 9 to 21 s, 92,245 pieces of 30 s data	48 pieces of 30 min data	48 pieces of 30 min data	48 pieces of 30 min data	51,261 pieces of data ranging from 10 to 45 s	48 pieces of 30 min data, 23 pieces of 10 h data, 18 pieces of data ranging from 7 to 10 s, 6877 pieces of data ranging from 6 to 60 s
Parameter quantity/M		5.2	4.7	3.9	3.5	4.2	6.3	5.3
N	F1	0.7280	0.9861	0.9900	0.9850	0.9784	0.9774	0.9925
ST		-	-	-	-	-	0.9069	0.9243
SB		-	-	-	-	-	0.8977	0.8591
PAC		0.8070	0.8199	0.8400	0.7910	0.6715	0.6946	0.9621
AF		0.9200	0.9449	-	-	-	0.9077	0.9888
PVC		0.8780	0.8439	0.9700	0.9340	0.9280	0.7184	0.9916
Overall accuracy		-	-	0.9800	0.7520	0.6120	0.9550	0.9855
Overall F1-score		0.8250	0.8852	0.9333	0.9033	0.8593	0.8504	0.9531

## Data Availability

The dataset used in the article is derived from a publicly available dataset. The download link is as follows. MIT-BIH Arrhythmia Database (accessed on 10 December 2025): https://physionet.org/content/mitdb/1.0.0/; MIT-BIH Atrial Fibrillation Database (accessed on 10 December 2025): https://physionet.org/content/afdb/1.0.0/; CODE Dataset (accessed on 10 December 2025): https://zenodo.org/records/4916206.

## Abbreviations

The following abbreviations are used in this manuscript:

ECG	Electrocardiogram
CNNs	Convolutional Neural Networks
RNNs	Recurrent Neural Networks
LSTM	Long and Short-term Memory Network
BiLSTM	Bidirectional Long and Short-term Memory Network
AAMI	Association for the Advancement of Medical Instrumentation
MHSA	Multi-Head Self-Attention Mechanism
CPSC 2018	the China Physiological Signal Challenge 2018 dataset
Db6	Daubechies6
N	Normal beat
ST	Sinus Tachycardia
SB	Sinus Bradycardia
PAC	Premature Atrial Contraction
AF	Atrial Fibrillation
PVC	Premature Ventricular Contraction
BN	Batch Normalization
ReLU	Rectified Linear Unit
TP	True Positive
FN	False Negative
FP	False Positive
TN	True Negative
ROC	Receiver Operating Characteristic
AUC	Area Under the Curve
FPR	False Positive Rate
TPR	True Positive Rate
PR	Precision-Recall
AUPR	Area Under the PR Curve
HRV	Heart Rate Variability
